# Differential detectability of pericyte and blood–brain barrier readouts across the Alzheimer’s disease clinical continuum: a systematic review and meta-analysis

**DOI:** 10.3389/fnagi.2026.1870764

**Published:** 2026-07-30

**Authors:** Wenchuan Wang, Hanying Xu, Tong Wu, Yibin Zhang, Mengqi Tao, Xuanheng Zhao, Jing Lu, Xiaolei Tang, Shuo Zhang, Ying Zhang, Hongmei Nan

**Affiliations:** 1 College of Traditional Chinese Medicine, Changchun University of Chinese Medicine, Changchun, China; 2 Department of Encephalopathy, The Affiliated Hospital of Changchun University of Chinese Medicine, Changchun, China; 3 Research Center of Traditional Chinese Medicine, The Affiliated Hospital of Changchun University of Chinese Medicine, Changchun, China

**Keywords:** Alzheimer’s disease, blood–brain barrier, mild cognitive impairment, pericytes, soluble platelet-derived growth factor receptor-β

## Abstract

**Background:**

In Alzheimer’s disease (AD), neurovascular unit and blood–brain barrier (BBB) abnormalities are assessed using biologically distinct readouts, often across separate cohorts, complicating interpretation across clinical stages and biomarker domains.

**Methods:**

We searched major databases (inception to February 7, 2026) for human observational studies quantifying cerebrospinal fluid (CSF) soluble platelet-derived growth factor receptor-*β* (sPDGFRβ), dynamic contrast-enhanced magnetic resonance imaging (DCE-MRI) *K*^trans^, or the CSF/serum albumin quotient (QAlb) in cognitively normal (CN), mild cognitive impairment (MCI), and AD dementia groups. Primary contrasts were MCI versus CN, AD dementia versus CN, and AD dementia versus MCI. Outcomes were analyzed separately using standardized mean differences (SMDs) in random-effects models with restricted maximum-likelihood estimation and Hartung–Knapp adjustment. Sensitivity analyses incorporated amyloid/AT (N)-informed biologic anchoring and evidence-tier restrictions where possible.

**Results:**

We included 24 cross-sectional studies (3,644 participants). Compared to CN, MCI showed higher CSF sPDGFRβ (SMD 0.38, 95% CI 0.03–0.73) and DCE-MRI *K*^trans^ (SMD 0.91, 95% CI 0.09–1.73; highly heterogeneous and based on five studies), whereas the difference for QAlb was smaller (SMD 0.19, 95% CI 0.01–0.37). Stricter etiologic restriction weakened the robustness of MCI-stage estimates, particularly for early AD-specific inferences in cohorts without consistent biomarker confirmation. Tier 1-only pooling was not feasible because biomarker-confirmed MCI-stage evidence was sparse. AD dementia versus CN comparisons showed higher values across all readouts: CSF sPDGFRβ (SMD 0.43, 95% CI 0.12–0.73), *K*^trans^ (SMD 0.90, 95% CI 0.56–1.25; based on four studies), and QAlb (SMD 0.28, 95% CI 0.10–0.45). AD dementia versus MCI comparisons showed no significant pooled differences for CSF sPDGFRβ or QAlb; *K*^trans^ pooling was precluded by insufficient data.

**Conclusion:**

Pericyte- and BBB-related readouts showed readout-specific, cross-sectional detectability patterns across the AD clinical continuum. *K*^trans^ findings should be considered preliminary because of the small evidence base, and MCI-stage estimates remain limited by etiologic uncertainty. Overall, CSF sPDGFRβ, DCE-MRI *K*^trans^, and QAlb appear related but non-interchangeable, and the present cross-sectional evidence should not be interpreted as demonstrating temporal priority, within-person progression, or head-to-head biomarker superiority.

**Systematic review registration:**

PROSPERO, CRD420251142518.

## Graphical abstract

**Figure:**
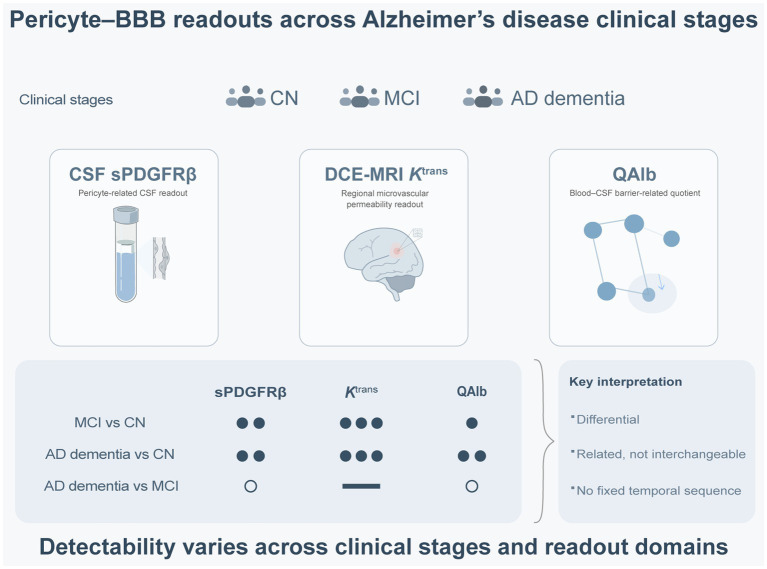
Relative cross-sectional detectability of CSF sPDGFRβ, DCE-MRI Ktrans, and QAlb across clinical-stage contrasts. Symbols indicate pooled effect-size magnitude: ● weak/low, ●● moderate, ●●● strong, ○ no significant difference, — insufficient data. Symbols reflect cross-sectional detectability only and do not imply temporal order or disease progression.

## 1 Introduction

Although Alzheimer’s disease (AD) is defined by amyloid-β (Aβ) and tau pathology (Jack et al., 2018), it develops in the context of brain aging, where neurovascular unit dysfunction, including vascular and blood–brain barrier (BBB) abnormalities, is increasingly recognized as relevant to AD pathophysiology (Sweeney et al., 2019). Neuroimaging and fluid biomarker findings suggest that BBB dysfunction may be detectable before or during early symptomatic stages (Nation et al., 2019) and may not be explained solely as a downstream consequence.

Preclinical evidence linking pericyte-related alterations with changes in barrier function and neuroinflammatory responses provides a biological rationale for focusing on the pericyte–BBB axis (Smyth et al., 2022). Pericytes are essential components of the neurovascular unit and play a central role in maintaining BBB integrity, regulating endothelial stability, and limiting abnormal permeability. In AD, pericyte dysfunction may contribute to BBB disruption, potentially interacting with vascular dysregulation, neuroinflammation, and impaired clearance of neurotoxic proteins. In human cohorts, however, this axis is assessed through readouts that capture distinct levels of pericyte- and barrier-related biology. Cerebrospinal fluid (CSF) soluble platelet-derived growth factor receptor-*β* (sPDGFRβ) has been used as a pericyte-related signal (Sagare et al., 2015; French et al., 2025), whereas dynamic contrast-enhanced MRI (DCE-MRI) *K*^trans^ is an imaging marker of regional microvascular permeability or leakage (Barisano et al., 2022); and the CSF/serum albumin quotient (QAlb), by contrast, is better interpreted as a relatively global, albumin quotient-based marker of barrier dysfunction rather than a direct whole-brain readout of BBB integrity (Skillbäck et al., 2017). These indicators reflect different, only partly overlapping aspects of the pericyte–BBB axis rather than interchangeable evidence of a single process.

Studies of these readouts remain fragmented and methodologically heterogeneous. In particular, CSF sPDGFRβ, DCE-MRI *K*^trans^, and QAlb have rarely been compared systematically across cognitively normal (CN), mild cognitive impairment (MCI), and AD dementia groups. As a result, the stage-related significance and detectability of these readouts across the AD clinical spectrum remain unclear (Heye et al., 2016; Thrippleton et al., 2019). Interpretation is especially challenging at the MCI stage, where clinically defined cohorts may include mixed etiologies, while CN groups may include individuals with preclinical AD or other subclinical cerebrovascular abnormalities (Albert et al., 2011; Sperling et al., 2011). Apparent inconsistency across studies may therefore reflect differences in diagnostic precision and level of measurement rather than simply the presence or absence of barrier dysfunction.

We conducted a systematic review and meta-analysis of three pericyte–BBB axis readouts—CSF sPDGFRβ, DCE-MRI *K*^trans^, and QAlb—across clinically relevant contrasts within the AD clinical continuum, involving CN, MCI, and AD dementia groups. Where available, we also incorporated sensitivity analyses based on biologically anchored MCI definitions and amyloid-informed cohort characterization to assess etiologic specificity. Our aim was to examine whether abnormalities along the pericyte–BBB axis show stage-sensitive, level-dependent detectability across the AD clinical continuum, without assuming a fixed temporal cascade or treating these readouts as interchangeable measures of a single process.

## 2 Methods

### 2.1 Study design and protocol registration

This systematic review and meta-analysis synthesized human observational evidence on pericyte- and BBB-related readouts across the Alzheimer’s disease (AD) continuum, focusing on three quantitatively extractable and biologically distinct outcomes: cerebrospinal fluid (CSF) soluble platelet-derived growth factor receptor-β (sPDGFRβ), dynamic contrast-enhanced MRI (DCE-MRI) *K*^trans^, and the CSF/serum albumin quotient (QAlb). The protocol was prospectively registered in PROSPERO (registration number: CRD420251142518). Compared with the registered protocol, several methodological refinements were made to improve feasibility, comparability, and interpretability without changing the core review question. These refinements were made after completion of the database search and title/abstract screening, during full-text assessment and data extraction, and before final quantitative pooling. Specifically, the final quantitative synthesis focused on CSF sPDGFRβ, DCE-MRI *K*^trans^, and QAlb; plasma-only and post-mortem measures were not combined quantitatively with these readouts, and clinical-stage contrasts were used as the primary analytic framework because biomarker-defined AD-continuum groups were not consistently available across studies. These refinements are summarized in Supplementary Table S3. Reporting followed the PRISMA 2020 statement and checklist (Page et al., 2021).

Eligible designs included human observational studies, such as cross-sectional, case–control, and cohort studies. However, all studies included in the review and meta-analysis ultimately contributed only cross-sectional between-group comparisons. Animal studies, *in vitro* experiments, and purely methodological reports were excluded. Eligibility criteria, grouping rules, and analytic procedures were finalized before quantitative data synthesis. Amyloid-β (Aβ) or AT (N) biomarker information, when available, was incorporated into etiologic characterization, evidence-tier classification, and sensitivity analyses rather than used as a mandatory requirement for primary pooling (Jack et al., 2018; Dubois et al., 2021). Study samples and analytic comparison units were further classified into prespecified evidence tiers reflecting etiologic certainty, as described in Section 2.6. The three outcomes were analyzed separately as distinct readout levels along the pericyte–BBB axis and were not pooled across readout classes or treated as interchangeable measures of a single construct.

### 2.2 Literature search strategy

PubMed/MEDLINE, Embase, Web of Science Core Collection, and the Cochrane Library were searched from inception to February 7, 2026. The search strategy combined database-specific subject headings and free-text terms covering key concepts such as Alzheimer’s disease, mild cognitive impairment (MCI), cognitively normal/unimpaired status, pericytes, PDGFRβ, blood–brain barrier (BBB) dysfunction, *K*^trans^, and albumin quotient. Full search strategies for all databases are provided in the Supplementary materials, and the complete PubMed strategy is shown in Supplementary Table S1.

Searches were limited to English-language publications at the database level. Reference lists of relevant reviews and included studies were screened manually, and citation tracking, including Google Scholar, was performed to identify additional records. Database searching was performed by one reviewer; title/abstract screening and full-text assessment were performed independently by two reviewers.

### 2.3 Eligibility criteria and grouping framework

Studies were eligible if they enrolled adults and reported participants who could be classified into one or more clinically defined stages of the AD continuum—cognitively normal or cognitively unimpaired (CN/CU), mild cognitive impairment (MCI), and AD dementia—according to authors’ definitions and/or recognized clinical diagnostic frameworks (Albert et al., 2011; McKhann et al., 2011; Sperling et al., 2011; Dubois et al., 2021). Clinical-stage grouping served as the primary analytic framework. Aβ or AT (N) biomarker confirmation was not required for the primary clinical-stage meta-analyses; however, biomarker ascertainment and reported results were extracted, when available, and incorporated into etiologic characterization, evidence-tier classification, sensitivity analyses, and heterogeneity interpretation.

#### 2.3.1 CN/CU definition and contamination handling

CN/CU participants served as the primary reference group. Eligible control samples were described as cognitively normal, cognitively unimpaired, neurologically normal, or equivalent, and had no reported major central nervous system conditions likely to substantially affect BBB-related measures. When available, biomarker-defined Aβ-negative CN participants were prioritized as the cleanest reference group. Cognitively normal Aβ-positive individuals were not merged into overall CN estimates when separately extractable. Inseparable mixtures of Aβ-positive and Aβ-negative CN participants were retained in the primary analyses but flagged as potentially contaminated controls. Subjective cognitive decline (SCD)-only cohorts were excluded from the primary CN/CU reference group. No SCD cohorts were included in the final primary meta-analytic dataset.

#### 2.3.2 MCI definition and etiologic characterization

MCI was defined according to the study authors’ classification and could include amnestic or non-amnestic MCI, as well as prodromal AD or MCI due to AD when these labels were explicitly stated. To avoid etiologic over-assignment, MCI samples were classified as having AD-related etiologic support when they met at least one criterion: (1) explicit designation as prodromal AD or MCI due to AD (Albert et al., 2011; Dubois et al., 2021); (2) biomarker support from Aβ or AT (N); or (3) evaluation within an AD etiologic framework with documented exclusion of major non-AD causes. MCI samples not meeting these criteria were classified as MCI of uncertain etiology. Amnestic MCI was not assumed to represent AD in the absence of AD-related etiologic support. Studies centered on clearly non-AD MCI etiologies were excluded.

#### 2.3.3 AD dementia definition

AD dementia required recognized clinical diagnostic criteria, such as NIA-AA, NINCDS–ADRDA, or equivalent frameworks (McKhann et al., 1984; McKhann et al., 2011). Studies reporting all-cause dementia, unspecified dementia, or clearly non-AD dementias, including dementia with Lewy bodies, frontotemporal dementia, vascular dementia, or Parkinson’s disease dementia, were excluded from AD dementia pooling. Mixed dementia cohorts without a separable AD-dominant subgroup and without biomarker support were excluded from primary pooling and, when relevant, were considered only in sensitivity analyses or the narrative summary.

#### 2.3.4 Readouts and outcomes

Studies were required to report extractable quantitative data for at least one of three outcomes: (1) CSF sPDGFRβ, (2) DCE-MRI *K*^trans^, and/or (3) QAlb. CSF sPDGFRβ was not pooled with plasma sPDGFRβ or CSF/plasma sPDGFRβ ratios. Each outcome class was meta-analyzed separately.

#### 2.3.5 Additional exclusions

Case reports and studies without extractable quantitative group-level data were excluded. When required data were incompletely reported, corresponding authors were contacted; studies remained excluded from quantitative synthesis when the necessary data could not be obtained. Potentially overlapping cohorts were assessed using parent cohort name, recruiting institution, overlapping recruitment periods, author overlap, and similarity of sample characteristics and measurement procedures. If two or more reports were judged to represent the same or possibly overlapping cohort, only one dataset was retained for a given readout and contrast, with priority given to the report with the larger sample, more complete outcome data, and clearer clinical characterization.

### 2.4 Study selection

Records were imported into EndNote for deduplication and screened in Microsoft Excel. Two reviewers independently screened titles and abstracts and then assessed full texts for eligibility. Reasons for full-text exclusion were recorded. Disagreements were resolved through discussion, with adjudication by a third reviewer when required. The selection process is summarized in the PRISMA flow diagram.

### 2.5 Data extraction and harmonization

Data were extracted using a structured extraction form and checked by a second reviewer. Extracted study-level information included bibliographic characteristics, country, study design, recruitment setting, diagnostic framework, and Aβ/AT (N) biomarker ascertainment, when reported. For each study arm contributing to the analysis, we recorded the original group label, primary clinical-stage group (CN/CU, MCI, or AD dementia), secondary biomarker-defined subgroup information, when available, sample size, and flags related to control contamination, MCI etiologic uncertainty, and dementia etiologic specificity.

Each comparison unit was assigned an evidence tier (Tier 1–3) using predefined criteria for etiologic certainty. Extraction tables also recorded comparison-selection decisions, reasons for inclusion in or exclusion from the primary meta-analysis, and supporting notes or source quotations, where needed. Limited clinical severity information, such as Clinical Dementia Rating (CDR), was documented when reported but was not used for formal subgrouping or meta-regression. Information on vascular comorbidity and cerebrovascular burden was noted descriptively, when available, but was not extracted as a fully standardized covariate across all studies. Where reported, we also extracted APOE ε4 carrier status, sex distribution, age, cognitive severity measures, amyloid or AT (N) biomarker status, vascular comorbidities, cerebrovascular burden, MRI field strength, DCE-MRI region of interest, and pharmacokinetic model. These variables were tabulated descriptively and used to inform etiologic characterization, evidence-tier assignment, and interpretation of heterogeneity. They were not used for formal subgroup meta-analysis or meta-regression unless sufficient comparable data were available within a readout-specific and stage-specific contrast.

Readout-specific extraction included assay/platform information for CSF sPDGFRβ, region-of-interest (ROI) definitions and pharmacokinetic-model information for DCE-MRI *K*^trans^, and scaling details for QAlb. Because QAlb was reported using different scalings across studies, and these could not be reliably standardized across the full dataset, all pooled analyses were conducted using standardized mean differences.

To avoid unit-of-analysis errors, each study contributed at most one effect size per readout per meta-analytic contrast. For DCE-MRI *K*^trans^, when multiple ROIs were reported, one ROI was selected according to a priority order: hippocampus or medial temporal lobe, followed by posterior cingulate or precuneus, cortical gray matter, and white matter. When bilateral ROIs were reported without a combined value, left and right values were combined before meta-analysis. When multiple pharmacokinetic models were available for the same ROI, the authors’ primary model was used; if no primary model was stated, selection followed the order Tofts, extended Tofts, and Patlak. For multi-arm studies with more than one eligible case subgroup sharing a common control group, case subgroups were combined whenever possible to generate a single comparison. If direct combination was not possible, the control-group sample size was split evenly across comparisons to avoid double-counting (Chandler et al., 2019). A single study was not allowed to contribute the same control sample more than once to the same readout-specific pooled contrast.

When necessary, summary statistics were converted to means and standard deviations using predefined procedures. Means and standard deviations were derived from reported medians and interquartile ranges or from standard errors, when required (Wan et al., 2014; Luo et al., 2018; Higgins et al., 2019). For studies presenting eligible data only in graphical form, values were digitized using WebPlotDigitizer. Data conversion and digitization were performed by one reviewer and checked by a second reviewer. Corresponding authors were contacted when clarification or additional numerical data were required.

### 2.6 Evidence tier classification

Each study sample or analytic comparison unit was categorized into one of three evidence tiers reflecting etiologic certainty rather than methodological quality. Tier 1 represented biomarker-confirmed AD-spectrum ascertainment, defined by amyloid PET positivity, CSF AD biomarker evidence, AT (N)-based classification, or other explicit cohort-wide biomarker evidence supporting AD pathology. Tier 2 represented clinically likely AD-spectrum samples without biomarker confirmation but meeting the predefined minimum-information threshold for etiologic confidence, including recognized AD diagnostic criteria together with sufficient exclusion of major non-AD causes and/or relevant clinical or imaging information supporting an AD-spectrum interpretation. For MCI samples, Tier 2 required more than a syndrome-level MCI label; supportive information could include explicit classification as prodromal AD or MCI due to AD, amnestic-predominant presentation within an AD diagnostic framework, documented exclusion of major non-AD causes, or available biomarker information that was incomplete but directionally supportive of AD-spectrum disease. Amnestic MCI alone was not treated as definitive evidence of AD etiology when no additional etiologic support was provided. Tier 3 represented etiology-uncertain samples, typically based only on syndrome-level classification without biomarker confirmation or sufficiently detailed etiologic anchoring. This category included MCI or dementia groups for which the available report did not provide enough information to confirm AD-specific etiology or to exclude major non-AD or mixed pathologies.

Tier classification was applied to all analytic comparison units included in the review. In quantitative analyses, tier-based restriction was implemented as a sensitivity analysis limited to Tier 1 plus Tier 2 evidence, allowing the primary clinical-stage analyses to retain broader coverage while testing the robustness of more etiologically anchored evidence. A more restrictive Tier 1-only synthesis was prespecified but could not be performed because of insufficient data. Specifically, too few biomarker-confirmed studies were available within most readout-specific and stage-specific contrasts to support stable Tier 1-only pooling.

Tier assignment was performed by reviewer consensus using the predefined criteria and supporting extraction notes. Because tier assignment was not conducted as a fully independent duplicate rating process, formal inter-rater reliability statistics were not calculated; this limitation is acknowledged in the Discussion.

### 2.7 Risk of bias assessment

Because all studies included in the review and meta-analysis were cross-sectional between-group comparisons, the 8-item Joanna Briggs Institute (JBI) Critical Appraisal Checklist for Analytical Cross-Sectional Studies was used as the primary critical appraisal tool (Moola et al., 2024). This checklist provided a standardized appraisal of methodological quality across studies. To complement this appraisal, we used a structured set of AD-specific interpretive domains tailored to the clinical and measurement heterogeneity of this literature. These domains were used to support etiologic and measurement-related interpretation rather than formal risk-of-bias judgment or a separate quality score. The assessed domains included the following: (1) clarity of diagnostic group definitions; (2) contamination risk in CN groups; (3) etiologic characterization of MCI; (4) specificity of AD dementia classification; (5) exclusion of major non-AD causes; (6) clarity of biomarker or imaging measurement procedures; (7) completeness of readout data; and (8) transparency of reporting and analysis.

Each study was evaluated using the JBI checklist, with supporting notes and source quotations recorded to enhance consistency and traceability. The AD-specific interpretive domains were considered alongside the JBI appraisal to aid interpretation of etiologic certainty, clinical group comparability, and readout-related heterogeneity. Risk-of-bias and interpretive appraisal were performed through reviewer consensus and informed both sensitivity analyses and narrative interpretation. A formal GRADE certainty assessment was not undertaken because the included evidence consisted of heterogeneous observational, cross-sectional, readout-specific comparisons with small numbers of studies in several key contrasts. Instead, certainty-related issues were addressed through JBI appraisal, evidence-tier classification, sensitivity analyses, and cautious interpretation of readout-specific robustness.

### 2.8 Statistical analysis

Primary meta-analyses were performed separately for each readout/outcome and organized by clinical-stage contrast: MCI versus CN, AD dementia versus CN, and AD dementia versus MCI, where data were sufficient. Sensitivity analyses incorporating available biologic anchoring information, including biologically anchored MCI definitions and amyloid-informed cohort characterization, were conducted when data permitted. Fully biomarker-defined validation meta-analyses based on Aβ status were prespecified but could not be meaningfully performed because comparable Aβ-defined datasets were too limited.

All pooled continuous outcomes were synthesized as standardized mean differences (Hedges, 1981) using random-effects models with restricted maximum-likelihood (REML) estimation (Deeks et al., 2019) and Hartung–Knapp adjustment (IntHout et al., 2014). Meta-analysis was performed when at least three independent studies were available for a given readout-specific contrast. Heterogeneity was assessed using Cochran’s Q, I^2^, and τ^2^. Ninety-five percent prediction intervals were estimated.

Sensitivity analyses were conducted, when data permitted, to examine the robustness of pooled estimates. These included the following: (1) restriction to Tier 1 plus Tier 2 evidence; (2) leave-one-out analyses; (3) exclusion of studies or comparison units with uncertain MCI etiology; (4) exclusion of mixed or etiologically uncertain dementia samples; (5) exclusion of studies requiring digitization of graphically reported values; and (6) assay-platform subgroup analyses, where relevant. Planned exclusion analyses for potentially contaminated or otherwise high-risk CN samples were not performed because of insufficient data. No meta-regression was conducted because the number of studies per readout-specific contrast was limited. Planned subgroup analyses or meta-regressions based on APOE ε4 status, sex, vascular comorbidity, cognitive severity, amyloid burden, MRI field strength, DCE-MRI ROI, or pharmacokinetic model were not undertaken because these variables were inconsistently reported and because the number of studies within each readout-specific and stage-specific contrast was too small to support reliable moderator analysis.

Exploratory funnel plots were inspected for readout-contrast pairs with sufficient numbers of studies, specifically for QAlb in the MCI versus CN and AD dementia versus CN comparisons. Formal tests for small-study effects were performed only for readout-contrast pairs including at least 10 studies. Therefore, Egger’s regression was conducted for the two QAlb contrasts that met this threshold (Egger et al., 1997; Higgins et al., 2024). All statistical tests were two-sided, with *α* = 0.05.

Meta-analytic calculations and sensitivity analyses were conducted in R version 4.4.2 (R Foundation for Statistical Computing, Vienna, Austria) using the meta (Balduzzi et al., 2019) and metafor (Viechtbauer, 2010) packages. Forest plots were prepared for presentation using Review Manager Web (RevMan Web).

## 3 Results

### 3.1 Study selection

**Figure 1:**
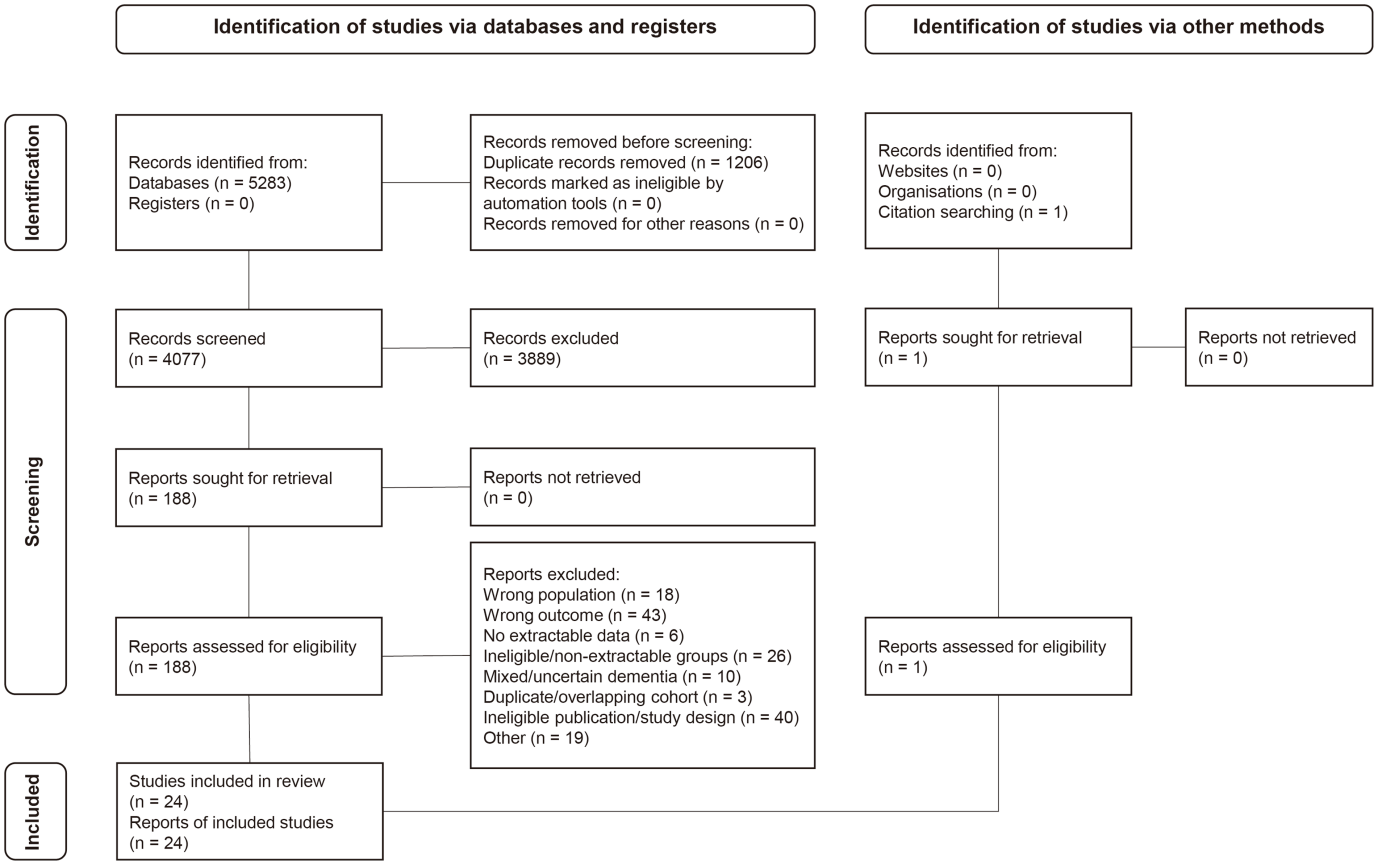
PRISMA flow diagram.

A total of 5,283 records were identified through database searching. After removal of 1,206 duplicates, 4,077 records underwent title and abstract screening, and 188 reports were retrieved for full-text assessment. Additionally, 1 report identified through citation searching was retrieved for full-text assessment. Therefore, 189 reports were assessed for eligibility. Ultimately, 24 studies were included in the systematic review and quantitative synthesis. The main reasons for full-text exclusion (*n* = 165) were ineligible outcome (*n* = 43), ineligible publication or study design (*n* = 40), ineligible or non-extractable groups (*n* = 26), ineligible or irrelevant population (*n* = 18), other reasons (*n* = 19), mixed or uncertain dementia etiology (*n* = 10), no extractable data (*n* = 6), and duplicate or overlapping cohorts (*n* = 3). No reports were unavailable for retrieval (Figure 1).

### 3.2 Study characteristics, evidence structure, and risk of bias

The 24 included studies comprised 3,644 unique participants across the clinical continuum. These studies reported data on one or more of the three prespecified readouts along the pericyte–BBB axis: CSF sPDGFRβ, DCE-MRI *K*^trans^, and QAlb. Ten studies reported CSF sPDGFRβ, 8 reported DCE-MRI *K*^trans^, and 14 reported QAlb. Study coverage across clinical stages varied, with some studies contributing only two-group contrasts and others spanning CN, MCI, and AD dementia. Study characteristics and evidence structure are summarized in Table 1.

**Table 1:** Characteristics of included studies and evidence structure.

Study and country	Outcomes	Groups, *n*	Age and sex (% female)	AD ascertainment/biomarker support (evidence tier)	APOE ε4 status	Key measurement details
Chalbot et al. (2011) Sweden; Norway; USA	QAlb	CN:21 MCI:NA AD:54	CN: 59.4 ± 9.2 (43.0%) AD:69.3 ± 4.3 (46.0%)	Clinical AD (NINCDS-ADRDA); HC; no cohort-wide Aβ/AT (N) confirmation (Tier 2)	Reported in subset	CSF/serum albumin ratio
Contreras et al. (2024) USA	CSF sPDGFRβ	CN:67 MCI:22 AD:NA	CN: 64.57 ± 10.4 (53.8%) MCI:65.5 ± 15.4 (50.0%)	Early cognitive impairment by CDR; CN; no cohort-wide biomarker-confirmed AD classification (Tier 3)	Reported by group	Quantitative Western blot
De Kort et al. (2022) Netherlands; USA	CSF sPDGFRβ QAlb	CN:32 MCI:19 AD:40 CN:10 MCI:13 AD:17	CN: 61.71 ± 8.3 (43.0%) MCI: 73.5 ± 6.0 (43.0%) AD: 71.4 ± 8.8 (72.5%) CN: 63.0 ± 10.0 (50.0%) MCI: 72.0 ± 5.0 (46.1%) AD: 71.0 ± 8.0 (52.9%)	Clinical amnestic MCI/AD (Petersen/NINCDS-ADRDA); controls; AD biomarker subgroup available (Tier 2)	Reported by group	ELISA CSF/serum albumin ratio
Gan et al. (2024) China	DCE MRI *K*^trans^	CN:24 MCI:NA AD:29	CN: 68.9 ± 5.5 (58.3%) AD:71.7 ± 7.3 (48.3%)	Clinical AD (NIA-AA); HC; plasma Aβ data only (Tier 2)	Reported overall only	3 T; Patlak; hippocampus MTL
Janelidze et al. (2017) Sweden	QAlb	CN:65 MCI:35 AD:75	CN: 75.0 ± 6.0 (64.6%) MCI:75.0 ± 8.0 (65.7%) AD:76.0 ± 7.0 (68.0%)	Clinical AD (NINCDS-ADRDA); HC; no cohort-wide amyloid confirmation (Tier 2)	Reported by group	CSF/serum albumin ratio
Kim et al. (2015) Korea	QAlb	CN:25 MCI:NA AD:32	CN: 60.6 ± 6.3 (76.0%) AD:60.0 ± 6.2 (71.9%)	Clinical sEOAD (NIA-AA); controls; partial CSF biomarker support (Tier 2)	Reported by group	CSF/serum albumin ratio
Le Bastard et al. (2009) Belgium	QAlb	CN:15 MCI:24 AD:37	CN: 66.0 ± 16.3 (62.1%) MCI:75.9 ± 7.5 (46.2%) AD:82.0 ± 5.9 (65.3%)	Clinical AD/MCI (strict clinical criteria/Petersen); controls; no cohort-wide biomarker confirmation (AD Tier 2; MCI Tier 3)	NR	CSF/serum albumin ratio
Lin et al. (2021) USA	QAlb	CN:17 MCI:17 AD:NA	CN: 70.6 ± 5.4 (64.7%) MCI: 68.1 ± 8.9 (47.1%)	Clinical MCI (NIA-AA framework); cognitively normal controls; CSF AD biomarkers assayed in subset (Tier 3)	Reported by group	CSF/serum albumin ratio
Llorens et al. (2015) Germany	QAlb	CN:34 MCI:23 AD:90	CN: 66.0 ± 9.5 (44.1%) MCI:69.5 ± 7.8 (39.1%) AD:72.8 ± 10.9 (77.8%)	Clinical AD/MCI (ICD-10/neuropsychological evaluation); controls; core CSF biomarkers assayed (AD Tier 2; MCI Tier 3)	NR	CSF/serum albumin ratio
Lv et al. (2023) China	CSF sPDGFRβ QAlb	CN:27 MCI:48 AD:83	CN: 60.7 ± 6.6 (44.4%) MCI: 63.2 ± 9.0 (60.4%) AD: 64.7 ± 7.6 (68.7%)	Clinical AD/MCI (NIA-AA); CN controls; all AD A+, mixed MCI amyloid status (AD Tier 1; MCI Tier 3)	Reported by group	ELISA CSF/serum albumin ratio
Miners et al. (2019) Sweden	CSF sPDGFRβ	CN:39 MCI:NA AD:39	CN: 68.2 ± 11.9 (41.0%) AD: 76.2 ± 6.1 (36.0%)	Biomarker-defined profiles; Aβ + and tau+ required for AD, normal levels for CN (Tier 1)	NR	ELISA
Miners et al. (2025) USA	CSF sPDGFRβ QAlb	CN:25 MCI:25 AD:25	CN: 69.5 ± 5.3 (60.0%) MCI: 71.7 ± 6.9 (48.0%) AD: 73.0 ± 9.1 (40.0%)	Clinical AD/MCI; CU controls; CSF Aβ/tau-defined with PET Aβ/tau available (Tier 1)	Reported by group	ELISA CSF/serum albumin ratio
Montagne et al. (2015) USA	CSF sPDGFRβ DCE MRI *K*^trans^	CN:14 MCI:17 AD:NA CN:18 MCI:20 AD:NA	CN (18): 73.2 ± 10.6 (55.6%) MCI (21): 72.0 ± 8.5 (52.4%)	Clinical MCI (CDR 0.5); NCI controls; no cohort-wide Aβ/AT (N) confirmation (Tier 3)	NR	Unclear; hippocampus MTL
Moon et al. (2021) Korea	DCE MRI *K*^trans^	CN:33 MCI:33 AD:NA	CN: 65.7 ± 6.4 (66.7%) MCI:65.9 ± 6.6 (63.6%)	Clinical MCI (Petersen/NINCDS-ADRDA-based); NC; APOE4 genotyped only (Tier 3)	Reported in subset	3 T; Patlak; hippocampus MTL
Musaeus et al. (2020) Denmark	QAlb	CN:34 MCI:NA AD:289	CN: 65.94 ± 7.53 (58.8%) AD:72.64 ± 8.9 (55.7%)	Clinical AD (NIA-AA/NINCDS-ADRDA); HC; no cohort-wide biomarker confirmation (Tier 2)	NR	CSF/serum albumin ratio
Nation et al. (2019) USA	CSF sPDGFRβ DCE MRI *K*^trans^	CN:82 MCI:65 AD:NA CN:44 MCI:32 AD:NA	Not reported	Early cognitive dysfunction (CDR-based); CN; Aβ/pTau subgrouping available (Tier 3)	Adjusted for APOE ε4; frequency NR	Quantitative Western blot; hippocampus MTL; Patlak
Ott et al. (2018) USA	QAlb	CN:21 MCI:8 AD:11	CN: 46.0 ± 11.0 (42.8%) MCI:69.0 ± 7.0 (12.5%) AD:67.0 ± 10.0 (54.5%)	Clinical AD/MCI (NINCDS-ADRDA/MCI Working Group); controls; CSF Aβ/pTau assayed (AD Tier 2; MCI Tier 3)	NR	CSF/serum albumin ratio
Preis et al. (2024) Germany	CSF sPDGFRβ DCE MRI *K*^trans^ QAlb	CN:44 MCI:18 AD:15 CN:44 MCI:21 AD:26 CN:13 MCI:10 AD:10	CN (44): 73.3 ± 5.8 (32.0%) MCI (21): 74.5 ± 7.0 (43.0%) AD (26): 74.5 ± 8.3 (58.0%)	Mixed-etiology MCI; NC; biomarker-defined AD/non-AD subgrouping available (AD Tier 2; MCI Tier 3)	Reported in subset	ELISA; Patlak; hippocampus MTL; CSF/serum albumin ratio
Skillbäck et al. (2017) Sweden	QAlb	CN:292 MCI:NA AD:796	CN: 73.0 ± 5.0 (60.0%) AD:72.4 ± 8.2 (66.2%)	Clinical AD (SveDem/ICD-10); HC; partial biomarker characterization only (Tier 2)	NR	CSF/serum albumin ratio
Sun et al. (2025) China	DCE MRI *K*^trans^	CN:55 MCI:102 AD:NA	CN: 70.24 ± 3.44 (41.8%) MCI:71.3 ± 4.5 (41.2%)	Clinical MCI (Petersen); HC; no biomarker-confirmed cohort classification (Tier 3)	NR	Patlak; hippocampus MTL
Sweeney et al. (2020) USA	CSF sPDGFRβ	CN:59 MCI:35 AD:NA	CN: 77.45 ± 6.6 (59.0%) MCI: 75.6 ± 5.9 (36.0%)	Early cognitive impairment/mild dementia (CDR-based ADRC cohort); cognitively normal controls; no cohort-wide Aβ/AT (N) confirmation (Tier 3)	Reported by group	SD-ECL immunoassay
Vrillon et al. (2025) France	CSF sPDGFRβ QAlb	CN:23 MCI:84 AD:NA	CN: 62.79 ± 9.10 (39.0%) MCI: 69.99 ± 10.53 (37.0%)	Biomarker-defined AD/non-AD cohort; NC; mixed MCI/dementia etiologies (Tier 3)	Reported overall only	ELISA CSF/serum albumin ratio
Wen et al. (2025) China	DCE MRI *K*^trans^	CN:26 MCI:NA AD:38	Age not reported as mean ± SD CN: (61.5%) AD: (47.4%)	Clinical AD (NINCDS-ADRDA); HC; partial biomarker data (Tier 2)	NR	3 T; Patlak; hippocampus MTL
Xu et al. (2025) China	DCE MRI *K*^trans^	CN:22 MCI:NA AD:42	CN: 68.7 ± 5.6 (63.6%) AD: 72.2 ± 6.9 (47.6%)	Clinical AD (NIA-AA); HC; no cohort-wide Aβ/AT (N) confirmation (Tier 2)	Reported by group	3 T; Model 3 tracer kinetic model; hippocampus MTL

Age is presented as mean ± SD and sex as % female unless otherwise stated. Sample sizes refer to the analytic subset for the listed outcome and may differ across outcomes within the same study. Evidence tiers indicate etiologic certainty rather than methodological quality: Tier 1, biomarker-confirmed AD-spectrum; Tier 2, clinically likely AD-spectrum without cohort-wide biomarker confirmation; Tier 3, etiology-uncertain. APOE ε4 status and key measurement details are summarized as reported in the original studies. AD, Alzheimer’s disease; CN, cognitively normal; MCI, mild cognitive impairment; APOE, apolipoprotein E; DCE-MRI, dynamic contrast-enhanced MRI; K^trans^, volume transfer constant; QAlb, CSF/serum albumin quotient; CSF, cerebrospinal fluid; sPDGFRβ, soluble platelet-derived growth factor receptor-β; NR, not reported; NA, not available or not applicable.

**Figure 2:**
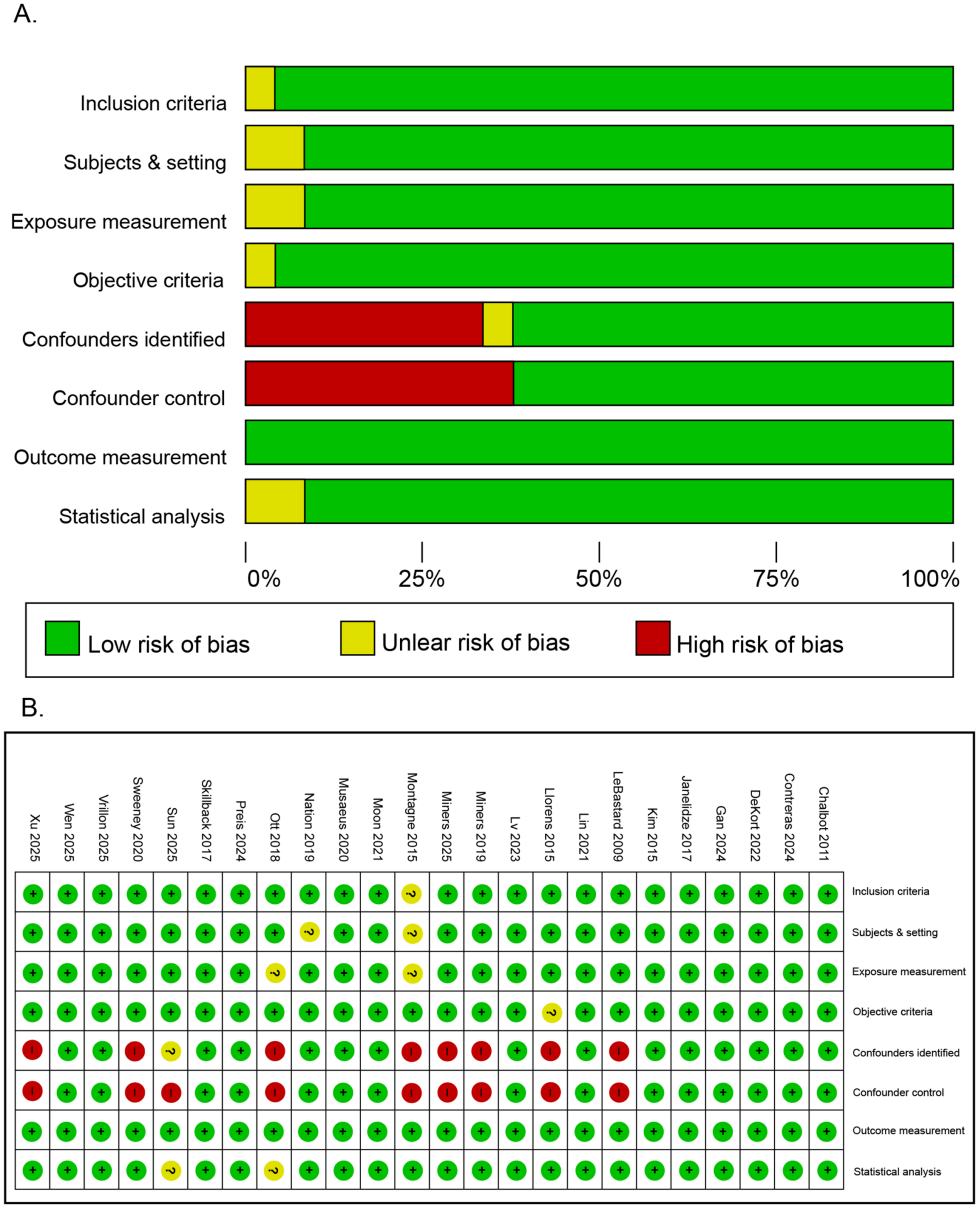
JBI critical appraisal of included studies **(A)** Summary plot based on the 8-item analytical cross-sectional checklist. Proportions of studies judged as low, unclear, or high risk across the eight JBI items. **(B)** Traffic-light plot based on the 8-item analytical cross-sectional checklist. Item-level JBI critical appraisal judgments for each included study. For visualization, JBI item responses were mapped as follows: Yes, low risk; No, high risk; Unclear, unclear risk.

Amyloid or AT (N) information was available for a substantial subset of the included studies, allowing biologic anchoring and tier-based characterization of part of the evidence base. Risk of bias based on the JBI critical appraisal is summarized in Figure 2. Across the included studies, methodological concerns more often related to clinical group definition and etiologic characterization than to biomarker or imaging measurement procedures. Common issues included insufficient description of MCI etiology, limited characterization of CN groups with respect to biomarker status or potential contamination, incomplete exclusion or reporting of major non-AD causes, and incomplete reporting of readout data in some studies. The overall evidence structure across readouts, stage coverage, biologic anchoring, and tier assignment is shown in Supplementary Figure S1.

APOE ε4 status was variably reported, ranging from diagnostic-group-specific frequencies to overall or subset-level descriptions, covariate adjustment only, or no reporting. Vascular comorbidities, cerebrovascular burden, cognitive severity, and amyloid or AT (N) characterization were also not reported in a uniform manner across cohorts. In addition, measurement-related features differed across readouts, including DCE-MRI field strength, ROI definition, pharmacokinetic modeling approach, CSF sPDGFRβ assay platform, and QAlb scaling or reference conventions. These variables were therefore tabulated descriptively where available and used to inform the interpretation of heterogeneity. However, because reporting was incomplete and the number of studies within most readout-specific and stage-specific contrasts was limited, these factors did not provide a sufficiently consistent basis for formal subgroup analysis or meta-regression.

### 3.3 CSF sPDGFRβ

**Figure 3:**
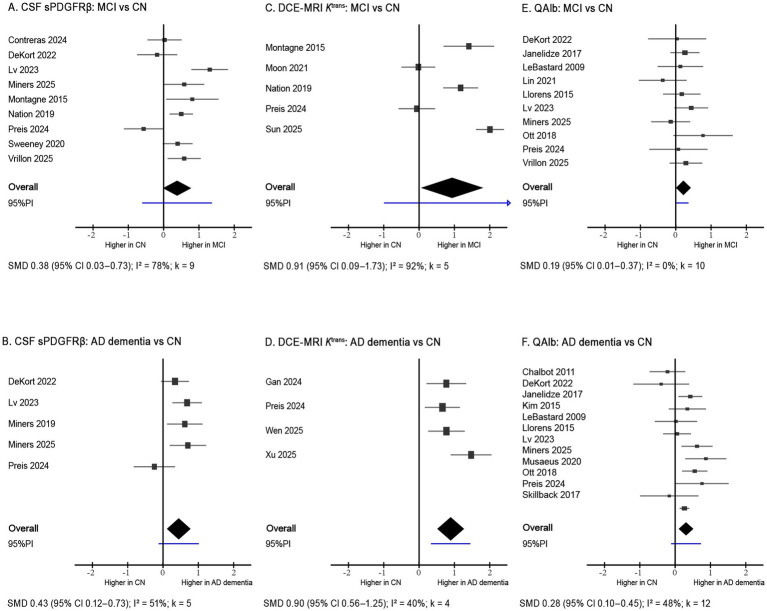
Main meta-analysis forest plots across the three readouts. CSF sPDGFRβ, MCI vs. CN; CSF sPDGFRβ, AD dementia vs. CN; DCE-MRI *K*^trans^; MCI vs. CN; DCE-MRI *K*^trans^, AD dementia vs. CN; QAlb, MCI vs. CN; QAlb, AD dementia vs. CN. **(A,C,E)** show mild cognitive impairment (MCI) versus cognitively normal (CN) comparisons, and **(B,D,F)** show Alzheimer’s disease (AD) dementia versus CN comparisons. Readouts are CSF soluble platelet-derived growth factor receptor-β (sPDGFRβ) **(A,B)** DCE-MRI volume transfer constant (*K*^trans^) **(C,D)** and the CSF/serum albumin quotient (QAlb) **(E,F)**. Squares indicate study-specific standardized mean differences (SMDs), with horizontal lines indicating 95% confidence intervals (CIs); square size reflects study weight. Diamonds indicate pooled random-effects estimates, and the separate horizontal lines beneath the diamonds indicate 95% prediction intervals (PIs). Positive SMDs indicate higher values in the symptomatic group than in CN. All panels are shown on the same *x*-axis scale; in **(C)**, the upper bound of the 95% PI extends beyond the plotted range and is indicated by an arrow. Summary statistics below each panel include the pooled SMD with 95% CI, *I*^2^, and *k*.

In the MCI versus CN contrast (9 studies), CSF sPDGFRβ levels were higher in the MCI group than in the CN group (SMD = 0.38, 95% CI 0.03 to 0.73, *p* = 0.03; I^2^ = 78%; Figure 3A). In the AD dementia versus CN contrast (5 studies), CSF sPDGFRβ levels were also higher in the AD dementia group than in the CN group (SMD = 0.43, 95% CI 0.12 to 0.73, *p* = 0.006; I^2^ = 51%; Figure 3B).

No statistically significant pooled difference was observed when the AD dementia and MCI groups were directly compared across 4 studies (SMD = 0.07, 95% CI -0.48 to 0.61, *p* = 0.81; I^2^ = 76%; Supplementary Figure S2A).

### 3.4 DCE-MRI Ktrans

In the MCI versus CN contrast (5 studies), DCE-MRI *K*^trans^ values were higher in the MCI group than in the CN group (SMD = 0.91, 95% CI 0.09 to 1.73, p = 0.03; I^2^ = 92%; Figure 3C). In the AD dementia versus CN contrast (4 studies), DCE-MRI *K*^trans^ values were higher in the AD dementia group than in the CN group (SMD = 0.90, 95% CI 0.56 to 1.25, *p* < 0.00001; I^2^ = 40%; Figure 3D).

Only 1 study reported the direct AD dementia versus MCI contrast; therefore, no pooled analysis was performed.

### 3.5 QAlb

For QAlb, the MCI versus CN contrast (10 studies) showed a small but statistically significant higher value in the MCI group (SMD = 0.19, 95% CI 0.01 to 0.37, *p* = 0.04; I^2^ = 0%; Figure 3E). In the AD dementia versus CN contrast (12 studies), QAlb was higher in the AD dementia group than in the CN group (SMD = 0.28, 95% CI 0.10 to 0.45, *p* = 0.002; I^2^ = 48%; Figure 3F).

Direct comparison of the AD dementia and MCI groups across 8 studies did not show a statistically significant pooled difference (SMD = 0.05, 95% CI -0.25 to 0.34, *p* = 0.76; I^2^ = 57%; Supplementary Figure S2B).

### 3.6 Cross-sectional stage-sensitive pattern across biomarker levels

**Figure 4:**
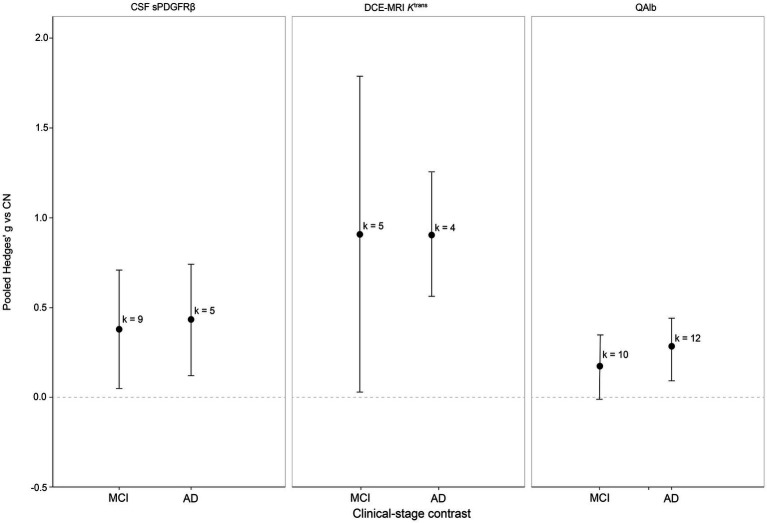
Cross-sectional stage-sensitive pattern across biomarker levels. Exploratory descriptive comparison of pooled effect estimates across clinical-stage contrasts. Pooled Hedges’ *g* values are shown for MCI and AD dementia groups relative to CN for CSF sPDGFRβ, DCE-MRI *K*^trans^, and QAlb. CN is not shown as a separate x-axis category but serves as the fixed reference group for all plotted estimates. Points indicate pooled estimates, and error bars indicate 95% confidence intervals. The horizontal zero line represents no difference relative to CN. Positive values indicate higher readout values in the symptomatic group than in CN. Estimates are presented for descriptive cross-sectional comparison only and should not be interpreted as evidence of within-person longitudinal change, temporal ordering, or a monotonic disease-stage trajectory. *k* denotes the number of contributing studies.

Figure 4 summarizes the pooled cross-sectional pattern across readout levels. Relative to the CN group, CSF sPDGFRβ levels and DCE-MRI *K*^trans^ values were higher in both the MCI versus CN and AD dementia versus CN contrasts. By contrast, QAlb showed a small pooled increase in the MCI versus CN contrast, with a clearer statistically significant increase in the AD dementia versus CN contrast. Direct pooled comparisons between the AD dementia and MCI groups were not statistically significant for CSF sPDGFRβ or QAlb, and comparable pooling was not feasible for DCE-MRI *K*^trans^. Overall, the pooled pattern suggested greater MCI-stage detectability for CSF sPDGFRβ and DCE-MRI *K*^trans^ than for QAlb, while direct symptomatic-stage separation remained limited by non-significant or insufficient AD dementia versus MCI evidence.

### 3.7 Sensitivity and robustness analyses

Sensitivity analyses for CSF sPDGFRβ and QAlb are shown in Supplementary Figures S3, S4, respectively. These analyses were performed only when at least three independent studies remained after restriction, consistent with the prespecified threshold for quantitative pooling.

For CSF sPDGFRβ, exclusion of studies with uncertain MCI etiology attenuated the MCI versus CN estimate and it no longer reached statistical significance (SMD = 0.33, 95% CI -0.11 to 0.76, *p* = 0.14; I^2^ = 59%), whereas exclusion of mixed or etiologically uncertain dementia yielded an AD dementia versus CN pooled estimate with a larger effect and no observed heterogeneity (SMD = 0.57, 95% CI 0.33 to 0.81, *p* < 0.00001; I^2^ = 0%). Tier 1 plus Tier 2 restriction was not presented as a separate pooled CSF sPDGFRβ analysis where the remaining study number was insufficient for stable readout-specific and stage-specific pooling.

For QAlb, the MCI versus CN contrast remained small and not statistically significant both after restriction to Tier 1 + 2 evidence (SMD = 0.21, 95% CI -0.06 to 0.48, *p* = 0.13; I^2^ = 0%) and after exclusion of studies with uncertain MCI etiology (SMD = 0.10, 95% CI -0.23 to 0.43, *p* = 0.55; I^2^ = 0%). In the AD dementia versus CN comparison, the corresponding study-restriction analysis did not materially alter the pooled estimate (SMD = 0.29, 95% CI 0.06 to 0.52, *p* = 0.01; I^2^ = 59%), whereas restriction to Tier 1 + 2 evidence yielded a positive but not statistically significant pooled estimate (SMD = 0.40, 95% CI -0.05 to 0.85, *p* = 0.08; I^2^ = 60%). Exclusion of mixed or etiologically uncertain dementia was not shown separately for QAlb because it affected only 1 study and did not materially alter the pooled estimate.

A Tier 1-only meta-analysis was not feasible for any readout-specific and stage-specific contrast because too few biomarker-confirmed studies remained after restriction. Therefore, Tier 1-only results were not presented as pooled estimates; instead, biomarker-confirmed evidence was incorporated through the evidence-tier classification and through Tier 1 plus Tier 2 sensitivity analyses where sufficient studies were available.

Additional restriction analyses were not pursued for DCE-MRI *K*^trans^ because pooled contrasts were based on small numbers of studies. The MCI versus CN comparison was already restricted to hippocampal Patlak-based measurements, and the AD dementia versus CN comparison included only 2 hippocampal studies, precluding informative further restriction. Accordingly, further tier-restricted, uncertain-MCI-exclusion, mixed-dementia-exclusion, ROI-specific, field-strength-specific, or pharmacokinetic-model-specific sensitivity analyses were not performed for DCE-MRI *K*^trans^. DCE-MRI findings were therefore interpreted cautiously and were not extended beyond the available study-level evidence.

Leave-one-out analyses for all pooled contrasts are presented in Supplementary Figure S5. Across outcomes, omission-specific estimates did not materially alter the overall pattern, although the MCI versus CN contrasts for CSF sPDGFRβ and DCE-MRI *K*^trans^ were more sensitive to omission of individual studies than the corresponding AD dementia versus CN contrasts. Where applicable, exclusion of estimates extracted from graphical data did not materially change the direction of the pooled findings. Formal moderator analyses based on APOE ε4 status, sex, vascular comorbidity, cognitive severity, amyloid burden, MRI field strength, ROI, or pharmacokinetic model were not conducted because these variables were inconsistently reported and sparsely distributed across readout-specific contrasts.

For QAlb, funnel plots were examined for the two contrasts with at least 10 studies (Supplementary Figure S6). Visual inspection did not suggest marked asymmetry, and Egger’s regression did not indicate evidence of small-study effects for either the MCI versus CN contrast (*p* = 0.78) or the AD dementia versus CN contrast (*p* = 0.44) (Supplementary Table S2). These analyses should nevertheless be interpreted cautiously given the limited number of studies available for small-study-effect assessment.

## 4 Discussion

The present meta-analysis suggests that pericyte- and blood–brain barrier-related readouts show stage-sensitive and level-dependent differences across the Alzheimer’s disease (AD) clinical continuum, but do not follow a simple monotonic pattern aligned with the severity of cognitive impairment. In pooled cross-sectional analyses, a pericyte-related CSF readout (sPDGFRβ) and an imaging index of regional permeability (DCE-MRI *K*^trans^) both showed higher values in MCI and AD dementia relative to cognitively normal individuals, although the MCI-stage *K*^trans^ estimates were substantially heterogeneous. In contrast, QAlb, a more global CSF/serum barrier-related quotient commonly used as an index of blood–CSF barrier permeability, showed only a small difference in MCI versus CN, with clearer alteration in AD dementia versus CN. Direct AD dementia-versus-MCI comparisons were not statistically significant for sPDGFRβ or QAlb, and sparse data precluded *K*^trans^ pooling. Taken together, the pooled pattern suggests that readout differences were more readily detected relative to cognitively normal individuals than across adjacent symptomatic stages, with QAlb showing a weaker MCI-stage signal than CSF sPDGFRβ or DCE-MRI *K*^trans^.

At the pericyte-related level, CSF sPDGFRβ showed a detectable MCI-versus-CN difference, consistent with its use as a pericyte-injury-related readout, although the robustness of this finding was limited. Notably, the pooled estimate attenuated and no longer reached statistical significance after restriction to studies with more etiologically anchored MCI samples. This suggests that the MCI-stage result was sensitive to cohort composition and became less robust after stricter etiologic restriction, rather than supporting CSF sPDGFRβ as an AD-specific staging marker. The AD dementia-versus-CN comparison was more consistent, whereas the direct AD dementia-versus-MCI comparison remained not statistically significant. Accordingly, although CSF sPDGFRβ may reflect pericyte injury, the current meta-analytic findings do not support a clear relationship between CSF sPDGFRβ levels and clinical-stage severity across adjacent symptomatic stages. This interpretation is also in line with the broader literature, in which CSF sPDGFRβ has been linked to age, BBB-related damage, and neuroinflammation more consistently than to core AD pathological staging (Nation et al., 2019; Divecha et al., 2025). Reporting of prespecified clinical, genetic, and methodological modifiers was uneven across the included studies. Between-study differences in assay platform, sample handling, and covariate adjustment may also have contributed to heterogeneity in CSF sPDGFRβ estimates, but these factors were not reported with sufficient consistency to support quantitative moderator analysis. A related issue is whether sPDGFRβ can be meaningfully assessed in plasma or other blood-based samples. Blood-based measurement would be clinically attractive because it is less invasive than lumbar puncture and may be more feasible for large-scale screening, repeated monitoring, and longitudinal cohort studies. However, plasma or serum sPDGFRβ should not be interpreted as directly interchangeable with CSF sPDGFRβ. Unlike CSF measurements, circulating sPDGFRβ may be influenced by peripheral vascular injury, endothelial or platelet-related sources, systemic inflammation, and pre-analytical differences in sample handling and assay platforms. Therefore, blood-based sPDGFRβ remains a promising but biologically less specific extension of the current CSF literature. Future studies should directly compare paired CSF and plasma sPDGFRβ, examine their relationships with DCE-MRI *K*^trans^ and QAlb, and determine whether blood-based sPDGFRβ provides information beyond established plasma AD biomarkers.

At the neuroimaging level, DCE-MRI *K*^trans^ showed clearer differences relative to cognitively normal individuals. As an imaging index of regional microvascular permeability, and unlike QAlb, which reflects barrier dysfunction more indirectly at a more global level, DCE-MRI *K*^trans^ may better capture the spatially localized features of BBB injury. In the current synthesis, statistically significant pooled differences were observed for both the MCI-versus-CN and AD dementia–versus-CN contrasts, which is consistent with the ability of DCE-MRI to detect subtle regional leakage. Importantly, the available MCI-stage literature was largely restricted to hippocampal Patlak-based analyses, meaning that the current evidence is weighted toward AD-vulnerable medial temporal regions rather than toward a uniform brain-wide pattern (van de Haar et al., 2016). This interpretation nevertheless remains provisional given the substantial heterogeneity of the MCI-stage pooled estimate, the methodological sensitivity of subtle-leakage DCE-MRI analyses, and the sparse direct evidence for AD dementia-versus-MCI comparisons. Differences in field strength, acquisition protocol, ROI definition, arterial input function handling, and pharmacokinetic modeling may have contributed to the variability of *K*^trans^ estimates across studies. However, because these features were incompletely and inconsistently reported, they could not be evaluated reliably as moderators.

Regarding the more global CSF/serum albumin quotient level, QAlb showed a more modest pattern of change across clinical-stage comparisons. It is better interpreted as an albumin quotient-based index commonly used to reflect blood–CSF barrier permeability, rather than as a direct or localized readout of capillary BBB integrity (Reiber, 2003; Pardridge, 2007). In this context, the small pooled difference in the MCI-versus-CN contrast may reflect either a genuinely modest group difference at the MCI stage or the limited ability of QAlb to capture spatially restricted barrier abnormalities that are more readily detected by regional DCE-MRI measures or pericyte-related CSF readouts (Engelhardt, 2008; Sweeney et al., 2018; Cheng et al., 2023). Because QAlb is influenced by age, sex, CSF turnover, and systemic or vascular factors, incomplete adjustment for these variables may have further reduced the specificity of QAlb for disease-stage-related differences, particularly at the MCI stage (Castellazzi et al., 2020; Musaeus et al., 2020). Although a statistically significant difference was observed in AD dementia versus CN, the AD dementia-versus-MCI comparison was not statistically significant and therefore does not support a simple linear escalation across adjacent clinical groups.

The limited robustness and high heterogeneity of some MCI-stage estimates—particularly the wide variance in regional *K*^trans^ and the attenuation of the sPDGFRβ signal after stricter restriction—are plausibly related to the diagnostic and etiologic heterogeneity inherent in primarily clinical-stage classification. Because amyloid or AT (N) biomarker information was not uniformly available across datasets, biologic anchoring was used for etiologic validation and sensitivity analyses rather than as the primary grouping rule (Vos et al., 2015; Petersen et al., 2018). As a result, clinically defined MCI groups could have included etiologically mixed cases, including those with vascular contributions, while some cognitively normal groups may also have included individuals with preclinical AD or subclinical cerebrovascular abnormalities (Jansen et al., 2015; Kapasi et al., 2017; Iadecola et al., 2019). This kind of etiologic admixture would be expected to affect MCI-stage comparisons more strongly than dementia-versus-CN contrasts, particularly when observed pooled effects are modest. At the same time, loss of robustness after stricter etiologic restriction may also reflect reduced precision in a smaller evidence base. Accordingly, variable robustness at the MCI stage should be interpreted in light of diagnostic precision and cohort composition, rather than as evidence against early-stage biological differences. APOE ε4 status and vascular comorbidity are particularly relevant in this context. Both are biologically plausible modifiers of pericyte- and BBB-related readouts, yet they were not reported in a sufficiently harmonized, diagnostic-group-specific manner across the included studies (Bell et al., 2012; Halliday et al., 2016). Therefore, the absence of APOE- or vascular-burden-specific pooled estimates should not be interpreted as evidence that these factors are unimportant. Rather, it reflects the limited granularity and consistency of the available evidence. Sex may also be an important moderator because it influences AD risk, cerebrovascular aging, BBB integrity, QAlb values, and CSF dynamics. However, sex-stratified sPDGFRβ, *K*^trans^, and QAlb results were rarely available, preventing quantitative moderator analysis. Future studies should report sex-stratified readout data and examine potential interactions among sex, APOE ε4 status, and vascular burden.

Synthesizing these findings, the current meta-analytic evidence suggests differential detectability across clinical-stage comparisons and across biologically non-equivalent readout levels. CSF sPDGFRβ, DCE-MRI *K*^trans^, and QAlb should not be treated as interchangeable readouts of a single uniform process. Instead, they are better interpreted as reflecting different levels of pericyte- and blood–brain barrier-related change: CSF sPDGFRβ is commonly interpreted as a pericyte-injury-related signal, DCE-MRI *K*^trans^ reflects regional microvascular permeability, and QAlb reflects more global albumin quotient-based blood–CSF barrier dysfunction (Tofts et al., 1999; Bowman et al., 2007; Cicognola et al., 2023). In the available cross-sectional literature, pooled group differences were larger in MCI-stage comparisons with sPDGFRβ and DCE-MRI *K*^trans^ than with QAlb (Zhao et al., 2015). However, these pooled cross-sectional profiles do not establish temporal ordering or a fixed monotonic sequence across individuals. Any inference about sequence or progression should remain cautious in light of cross-sectional study designs, incomplete etiologic anchoring in part of the literature, and readout-specific methodological differences (Bateman et al., 2012; Villemagne et al., 2013).

Several limitations should frame the interpretation of these findings. Heterogeneity remained substantial for some early-stage estimates, particularly for CSF sPDGFRβ and DCE-MRI *K*^trans^, where cohort composition and readout-specific methodological variations—including assay platforms, ROI definitions, and kinetic modeling approaches—were likely contributing factors (Wardlaw et al., 2013; Blennow and Zetterberg, 2018). Prediction intervals also supported cautious interpretation. Whereas confidence intervals describe uncertainty around the pooled mean effect, prediction intervals indicate the range of effects expected in a future comparable study. Wide prediction intervals, particularly those crossing the null, suggest that significant pooled estimates may not be consistently reproduced across future cohorts. This was especially relevant for early-stage sPDGFRβ and *K*^trans^ analyses, where heterogeneity was substantial and the number of studies was limited. In addition, the *K*^trans^ evidence base was relatively sparse, and the lack of sufficient AD dementia-versus-MCI data limited evaluation of direct separation across adjacent symptomatic stages. Amyloid and complete AT (N) biomarker characterization was not uniform across the included studies (Scheltens et al., 2021), and most of the available evidence was cross-sectional, precluding inference regarding within-person trajectories (Montagne et al., 2020). Because QAlb was reported using different scalings across studies, pooled synthesis relied on standardized mean differences; while this improved harmonization across datasets, it limited direct interpretation of absolute between-group differences. Finally, cross-readout comparisons in this review should be regarded as indirect rather than head-to-head, given that the three readout literatures differ in stage coverage, cohort selection, and measurement approaches, as well as in their underlying biological scope (Zlokovic, 2011; Daneman and Prat, 2015). Although APOE ε4 status, sex distribution, vascular comorbidities, cerebrovascular burden, cognitive severity, amyloid burden, MRI field strength, DCE-MRI ROI, pharmacokinetic model, assay platform, and QAlb scaling were prespecified as variables of interest, the number of studies within most readout-specific and stage-specific contrasts was small and reporting was incomplete. Formal subgroup analyses or meta-regression based on these factors would therefore have been statistically unstable and potentially misleading. These variables were consequently used to contextualize heterogeneity rather than to generate additional pooled estimates.

Future work would benefit from biologically anchored MCI cohorts, more harmonized imaging protocols, and integrated longitudinal studies that evaluate CSF sPDGFRβ, DCE-MRI *K*^trans^, and QAlb within the same participants as complementary pericyte-, regional permeability-, and albumin quotient-based barrier readouts (Erickson and Banks, 2013; Iturria-Medina et al., 2016). Such multimodal, within-subject designs would be particularly valuable for moving beyond cross-sectional detectability toward a more direct understanding of temporal and spatial relationships across readout levels. In a broader therapeutic context, better characterization of these distinct vascular and barrier-related vulnerabilities may inform future studies of treatment-associated vascular imaging abnormalities, such as amyloid-related imaging abnormalities (ARIA) (Salloway et al., 2022), although the present synthesis does not permit direct inference regarding anti-amyloid therapies (Greenberg et al., 2020). Overall, the available cross-sectional evidence supports a stage-sensitive and level-dependent pattern of detectability along the pericyte–BBB axis across the AD clinical continuum. These findings indicate that pericyte-related change, regional microvascular permeability, and albumin quotient-based barrier dysfunction should not be interpreted as interchangeable readouts of a single barrier-related process. However, the current evidence remains cross-sectional, heterogeneous, and limited in key direct between-stage comparisons, and therefore does not establish a fixed temporal cascade across readout levels.

## Data Availability

The original contributions presented in the study are included in the article/Supplementary material, further inquiries can be directed to the corresponding author/s.
