# Supplementary material for: Differential detectability of pericyte and blood–brain barrier readouts across the Alzheimer’s disease clinical continuum: a systematic review and meta-analysis
Source: Front Aging Neurosci. 2026 Jul 30;18:1870764. doi: 10.3389/fnagi.2026.1870764 (PMC13469275; doi:10.3389/fnagi.2026.1870764)
Supplement: Supplementary file 1 [file Table_1.DOCX]

Supplementary Material

Differential detectability of pericyte and blood-brain barrier readouts across the Alzheimer’s disease clinical continuum : a systematic review and meta-analysis

Wenchuan Wang • Hanying Xu • Tong Wu • Yibin Zhang • Mengqi Tao • Xuanheng Zhao • Jing lu • Xiaolei Tang • Shuo Zhang • Ying Zhang* • Hongmei Nan*

**Index**

| Figure | Content | Page |
| --- | --- | --- |
| Supplementary Figure 1 | Evidence-structure heatmap | 3 |
| Supplementary Figure 2 | Additional main meta-analysis forest plots for direct AD dementia vs MCI contrasts | 4 |
| Supplementary Figure 3 | Sensitivity analyses for CSF sPDGFRβ | 5 |
| Supplementary Figure 4 | Sensitivity analyses for Qalb | 6 |
| Supplementary Figure 5 | Leave-one-out analyses | 8 |
| Supplementary Figure 6 | Funnel plots for Qalb | 10 |
| Supplementary Table S1 | Full search strategies | 11 |
| Supplementary Table S2 | Egger’s regression results for QAlb | 13 |
| Supplementary Table S3 | Protocol refinements | 14 |

# 1 Supplementary Figure S1. Evidence-structure heatmap

**Supplementary Figure S2.**
Each row represents one included study. Columns summarize the availability of CSF sPDGFRβ, DCE-MRI *K*^trans^, and QAlb data; coverage of the clinical-stage groups CN, MCI, and AD; amyloid/AT(N) biomarker availability; and study-level evidence-tier classification. Colored cells indicate availability or inclusion, whereas gray cells indicate non-availability or non-inclusion.

# 2 Supplementary Figure S2. Additional main meta-analysis forest plots for direct AD dementia vs MCI contrasts

## 2.1 CSF sPDGFRβ: AD dementia vs MCI

## 2.2 QAlb: AD dementia vs MCI

# 3 Supplementary Figure S3. Sensitivity analyses for CSF sPDGFRβ

## 3.1 **CSF sPDGFRβ MCI vs CN (excluding uncertain MCI)**

## 3.2 CSF sPDGFRβ MCI vs CN (Tier 1+2 restricted)

## 3.3 CSF sPDGFRβ MCI vs CN (excluding digitized studies)

## 3.4 CSF sPDGFRβ MCI vs CN (assay-platform sensitivity)

## 3.5 CSF sPDGFRβ AD dementia vs CN (excluding uncertain MCI)

## 3.6 CSF sPDGFRβ AD dementia vs CN (excluding digitized studies)

# 4 Supplementary Figure S4. Sensitivity analyses for Qalb

## 4.1 QAlb MCI vs CN (excluding uncertain MCI)

## 4.2 QAlb MCI vs CN (Tier 1+2 restricted)

## 4.3 QAlb AD dementia vs CN (excluding uncertain MCI)

## 4.4 S4D.QAlb AD dementia vs CN (Tier 1+2 restricted)

# 5 Supplementary Figure S5. Leave-one-out analyses

.

# 6 Supplementary Figure S6. Funnel plots for Qalb

**Supplementary Figure S6.**
Funnel plots were generated for the two QAlb comparisons that included at least 10 studies. S7A, mild cognitive impairment (MCI) versus cognitively normal (CN); S7B, Alzheimer’s disease dementia (AD dementia) versus CN. Standardized mean differences (Hedges’ g) are plotted against standard error. Visual inspection was used to assess possible small-study effects.

# 7 Supplementary Table S1. Full search strategies

| **The search strategy for PubMed(record number: 754)** | |
| --- | --- |
| **Number** | **Search terms** |
| #1 | "Alzheimer Disease"[MeSH Terms] |
| #2 | "alzheimer*"[Title/Abstract] OR "preclinical AD"[Title/Abstract] OR "prodromal AD"[Title/Abstract] OR "mild cognitive impairment"[Title/Abstract] OR "MCI"[Title/Abstract] |
| #3 | #1 OR #2 265987 |
| #4 | "Pericytes"[MeSH Terms] |
| #5 | "pericyte*"[Title/Abstract] OR "platelet-derived growth factor receptor beta"[Title/Abstract] OR "PDGFRb"[Title/Abstract] OR "PDGFRbeta"[Title/Abstract] OR "sPDGFRbeta"[Title/Abstract] OR "soluble PDGFRbeta"[Title/Abstract] |
| #6 | #4 OR #5 14997 |
| #7 | "blood brain barrier"[MeSH Terms] |
| #8 | "blood brain barrier"[Title/Abstract] OR "BBB"[Title/Abstract] OR "dynamic contrast-enhanced"[Title/Abstract] OR "DCE-MRI"[Title/Abstract] OR "Ktrans"[Title/Abstract] OR "permeability constant"[Title/Abstract] |
| #9 | #7 OR #8 86194 |
| #10 | "albumin"[Title/Abstract] |
| #11 | "albumin quotient"[Title/Abstract] OR "albumin index"[Title/Abstract] OR "QAlb"[Title/Abstract] OR "CSF/serum albumin"[Title/Abstract] OR "CSF plasma albumin"[Title/Abstract] OR "blood-CSF barrier"[Title/Abstract] |
| #12 | #10OR #11 199509 |
| #13 | "cerebrospinal fluid"[Title/Abstract] OR "CSF"[Title/Abstract] OR "MRI"[Title/Abstract] OR "magnetic resonance imaging"[Title/Abstract] 743153 |
| #14 | #6 OR #9 OR #12 295650 |
| #15 | #3 AND #13 AND #14 1150 |
| #16 | #15AND ((humans[Filter]) AND (english[Filter])) 754 |
| **The search strategy for Cochrane library(record number: 108)** | |
| **Number** | **Search terms** |
| #1 | Alzheimer* OR "mild cognitive impairment" OR MCI OR "preclinical AD" OR "prodromal AD" |
| #2 | CSF OR "cerebrospinal fluid" OR MRI OR "magnetic resonance imaging" |
| #3 | pericyte* OR PDGFR OR PDGFRB OR "soluble PDGFR"  OR "blood brain barrier" OR BBB  OR "dynamic contrast enhanced" OR DCE OR Ktrans  OR "albumin quotient" OR "albumin index" OR QAlb |
| #4 | #1 AND #2 AND #3 108 |
|  |  |
|  |  |
| **The search strategy for Embase(record number: 2355)** | |
| **Number** | **Search terms** |
| #1 | **'alzheimer disease'**/exp OR **alzheimer***:ti,ab OR **'preclinical ad'**:ti,ab OR **'prodromal ad'**:ti,ab OR **'mild cognitive impairment'**:ti,ab OR **mci**:ti,ab |
| #2 | **'pericyte'**/exp OR **pericyte***:ti,ab OR **'platelet derived growth factor receptor beta'**:ti,ab OR **pdgfrb**:ti,ab OR **spdgfrb**:ti,ab OR **'soluble pdgfrb'**:ti,ab |
| #3 | **'blood brain barrier'**/exp OR **'blood brain barrier'**:ti,ab OR **bbb**:ti,ab OR **'dynamic contrast enhanced mri'**:ti,ab OR **'dce mri'**:ti,ab OR **ktrans**:ti,ab |
| #4 | **'albumin quotient'**:ti,ab OR **'albumin index'**:ti,ab OR **qalb**:ti,ab OR **'csf serum albumin'**:ti,ab OR **'csf plasma albumin'**:ti,ab OR **'blood csf barrier'**:ti,ab |
| #5 | (**'cerebrospinal fluid'**/exp OR **cerebrospinal**) AND **fluid**:ti,ab OR **csf**:ti,ab OR **mri**:ti,ab OR **'magnetic resonance imaging'**/exp |
| #6 | #2 OR #3 OR #4 |
| #7 | #1 AND #5 AND #6 |
| #8 | #7 AND **'human'**/de |
| #9 | #7 AND **'human'**/de AND [english]/lim 2355 |
| **The search strategy for WOS(record number: 2066)** | |
| **Number** | **Search terms** |
| #1 | TS=(Alzheimer) OR TS=(mild cognitive impairment) OR TS=(MCI) OR TS=(preclinical AD) OR TS=(prodromal AD) |
| #2 | TS=(cerebrospinal fluid) OR TS=(CSF) OR TS=(MRI) OR TS=(magnetic resonance imaging) |
| #3 | TS=(pericyte) OR TS=(PDGFRB) OR TS=(PDGFRb) OR TS=(platelet derived growth factor receptor) OR TS=(soluble PDGFR) |
| #4 | TS=(blood brain barrier) OR TS=(BBB) OR TS=(dynamic contrast enhanced) OR TS=(DCE) OR TS=(contrast enhanced MRI) OR TS=(Ktrans) OR TS=(K trans) |
| #5 | TS=(albumin quotient) OR TS=(albumin index) OR TS=(QAlb) OR TS=(blood CSF barrier) |
| #6 | **#3 OR #4 OR #5** |
| #7 | **#6 AND #1 AND #2 2081** |
| #8 | #7**and English (语种) 2066** |

# 8 Supplementary Table S2. Egger’s regression results for QAlb

| **Contrast** | **Test** | **t** | **df** | **p-value** | **Bias estimate** | **SE** |
| --- | --- | --- | --- | --- | --- | --- |
| MCI vs CN | Egger’s regression | -0.59 | 8 | 0.5705 | -0.7382 | 1.2481 |
| AD dementia vs CN | Egger’s regression | -0.08 | 10 | 0.9415 | -0.0562 | 0.7462 |

**Supplementary Table S2.**
Egger’s linear regression test for funnel plot asymmetry was performed for the two QAlb comparisons that included at least 10 studies: MCI versus CN and AD dementia versus CN. A two-sided P < 0.05 was considered suggestive of small-study effects. Results should be interpreted cautiously given the limited number of studies.

# 9 Supplementary Table S3. Key methodological refinements relative to the PROSPERO protocol

| **Domain / Step** | **Final review implementation** | **Timing / rationale** |
| --- | --- | --- |
| Review focus | Focused on CSF sPDGFRβ, DCE-MRI Ktrans, and QAlb across clinical-stage contrasts | After search & title/abstract screening, during full-text assessment, before final pooling; aligned with available human evidence |
| Plasma & post-mortem measures | Discussed narratively, not pooled quantitatively | During data extraction; preserved comparability across readouts |
| Grouping framework | Clinical-stage contrasts (CN/CU, MCI, AD dementia); biomarker info used for etiologic characterization and sensitivity analyses | During full-text assessment & data extraction; biomarker-defined groups not consistently reported |
| Risk-of-bias assessment | JBI critical appraisal for cross-sectional studies | Before RoB assessment; appropriate for included study designs |
| Sensitivity & moderators | Sensitivity analyses conducted where data permitted; potential moderators used to contextualize heterogeneity | After data extraction, before final interpretation; meta-regression not feasible due to small study numbers |
